# Extracellular vesicles released by senescent myoblasts affect recipient cells via miRNA-target interactions

**DOI:** 10.18632/aging.206379

**Published:** 2026-05-15

**Authors:** Michael Kamal, Donald Xhuti, Sophie Joanisse, Joshua P. Nederveen, Mark A. Tarnopolsky, Gianni Parise

**Affiliations:** 1 Exercise Metabolism Research Group, Department of Kinesiology, McMaster University, Hamilton L8S 4L8, Ontario, Canada; 2 Department of Pediatrics, McMaster University Health Sciences Centre, Hamilton L8N3Z5, Ontario, Canada; 3 MRC-Versus Arthritis Centre of Excellence for Musculoskeletal Ageing Research, School of Life Sciences, University of Nottingham, Nottingham NG7 2TQ, Nottinghamshire, United Kingdom

**Keywords:** cellular senescence, extracellular vesicles, myoblasts, miRNA, multi-omics

## Abstract

Cellular senescence is a state of irreversible cell cycle arrest accompanied by a robust Senescence-Associated Secretory Phenotype (SASP) that disrupts the local and systemic microenvironment. In skeletal muscle, aging is accompanied by the dysfunction of resident muscle stem cells, also known as satellite cells, as well as chronic, low-grade systemic inflammation, which is partially driven by the SASP. Extracellular vesicles (EVs) are emerging as potential mediators of SASP intercellular communication; however, little is known within the context of skeletal muscle senescence. Here, we observed that senescent myoblasts release SASP factors *in vitro* that reduce myotube diameter by approximately 30% (*p* < 0.05). EVs released by these cells contained miRNAs directly implicated in cellular senescence and anti-apoptotic signalling. We identified 22 differentially expressed miRNAs (FDR < 0.05; |FC| > 1.5) within senescent myoblast-derived EVs (SEVs), including miR-34a/b/c and miR-22. Furthermore, miR-301a-3p was revealed as a novel mediator of several genes related to senescence. SEV transfer onto myoblasts significantly upregulated the anti-apoptotic gene *Mdm2* (*p* < 0.05; 60% increase), a member of the senescence-associated super-enhancer family. Lastly, we integrated the miRNA gene target data with the transcriptomic profiles of adult (8mo) and geriatric (28mo) mouse gastrocnemius muscles and observed a significant overlap in gene regulation by SEV miRNAs during aging. Taken together, these results indicate that EVs and their miRNA cargo may be potential contributors to the SASP and senescence-associated signalling in skeletal muscle during advanced aging.

## INTRODUCTION

Aging is accompanied by the progressive decline of function across multiple body systems at the tissue, cellular, and molecular levels [1]. Within skeletal muscle, there is a substantial decrease in the content and function of the resident muscle stem cell (satellite cell) population in advanced age [2]. Recently, there has been a growing body of evidence implicating cellular senescence as a potential mediator of the aging-related dysfunction of satellite cells (MuSC) [3]. Senescence is the irreversible arrest of the cell cycle in response to extrinsic or intrinsic stressors [4]. Senescent cells can accumulate in aged muscle and decrease the prevalence of healthy, functional tissues [5]. Furthermore, these cells release detrimental factors that could promote musculoskeletal dyshomeostasis through a mechanism known as the Senescence-Associated Secretory Phenotype (SASP) [6]. Despite significant evidence linking cellular senescence to the aging process, the current understanding of the SASP in aging skeletal muscle cells remains limited.

SASP is a potent regulator of the microenvironment surrounding senescent cells. Factors secreted by these cells include inflammatory cytokines, chemokines, growth factors, and matrix metalloproteases, all of which disrupt homeostatic function in neighbouring cells [7]. Exactly how these factors are released from senescent cells and communicate with adjacent cells is now well understood. However, emerging evidence suggests that extracellular vesicles (EVs) are involved in senescence-associated intercellular communication by transporting specific cargo from senescent cells that promote SASP-like effects in other cells [8]. EVs encompass several populations of particles released by cells that can differ in their size, function, and formation [9]. Senescent cells are known to increase the secretion of EVs from several cell populations and across multiple types of senescence, including DNA damage-induced senescence [10–12]. Moreover, EVs contain proteins and microRNAs (miRNAs) that can promote senescence and the SASP [13]. MiRNAs are small, non-coding RNAs that regulate gene expression, primarily through post-transcriptional modifications [14, 15]. With aging, muscle-derived EVs contain elevated levels of miR-34a that can promote senescence in recipient bone marrow cells [16]. However, little else is known about the SASP-like effects of muscle-derived EVs.

Targeted approaches have investigated a select few miRNAs, such as miR-34a, within EVs isolated from muscle, but there has not been a microRNAome-based analysis of senescence-associated miRNAs within these particles. Moreover, there is very minimal evidence of these EVs having SASP-like characteristics. Therefore, we sought to investigate the SASP potential of miRNAs packaged within senescent myoblast-derived EVs. Here, we characterized the miRNA profile of EVs released by muscle cells *in vitro* and found that these small particles are packaged with miRNAs related to cellular senescence. We also reported several novel senescence-associated miRNAs and identified a promising senescent gene regulator using bioinformatics-based analyses. Furthermore, the present findings demonstrated that senescent-derived EVs promote anti-apoptotic signalling in recipient myoblasts and contain miRNAs that regulate a substantial subset of genes that are differentially expressed *in vivo* during advanced age.

## RESULTS

### Senescent myoblasts release factors that impair myotube differentiation

Senescent cells are known to release SASP factors that can influence their cellular environment [7]. We have previously established a model of myoblast senescence using the chemotherapeutic agent bleomycin, which acts by rapidly causing single- and double-stranded DNA breaks [17]. Treating C_2_C_12_ myoblasts with 14 μM bleomycin for 12h induces a senescence phenotype 48h after the damaging stimulus is removed, as evidenced by prominent SA-β-gal staining (Figure 1A), cell cycle arrest (Figure 1B), and elevated *Cdkn1a* mRNA expression (Figure 1C). To assess if these myoblasts produce SASP-like effects on neighbouring cells, we utilized transwell inserts with a 0.4-μm membrane pore which permits the exchange of extracellular factors between cell populations without direct cell-cell interaction (Figure 1D). Senescent (SEN) or non-senescent (NSEN) myoblasts cultured onto 0.4-μm transwell inserts were co-incubated with untreated myoblasts for 6 days in low-serum conditions to stimulate myotube formation. Over this period, NSEN inserts were replaced every 48h to minimize transwell myoblast fusion. After 6 days of differentiation, myotubes that were co-incubated with SEN myoblasts had elevated senescent gene expression, including *Trp53* (+18%; *p* < 0.05; Figure 1E) and *Cdkn1a* (+19%; *p* < 0.05; Figure 1F) mRNA. Notably, these myotubes also exhibited a 27% decrease in diameter (*p* < 0.05; Figure 1G–1H), a 30% reduction in surface area (*p* < 0.05; Figure 1I), and a 50% decrease in myonuclear index (*p* < 0.05; Figure 1J) relative to myotubes co-incubated with NSEN myoblasts. Taken together, these findings suggest that senescent myoblasts produce a secretome that elicits a SASP-like effect on adjacent cells, ultimately increasing senescent gene expression and impairing myotube formation.

**Figure 1:**
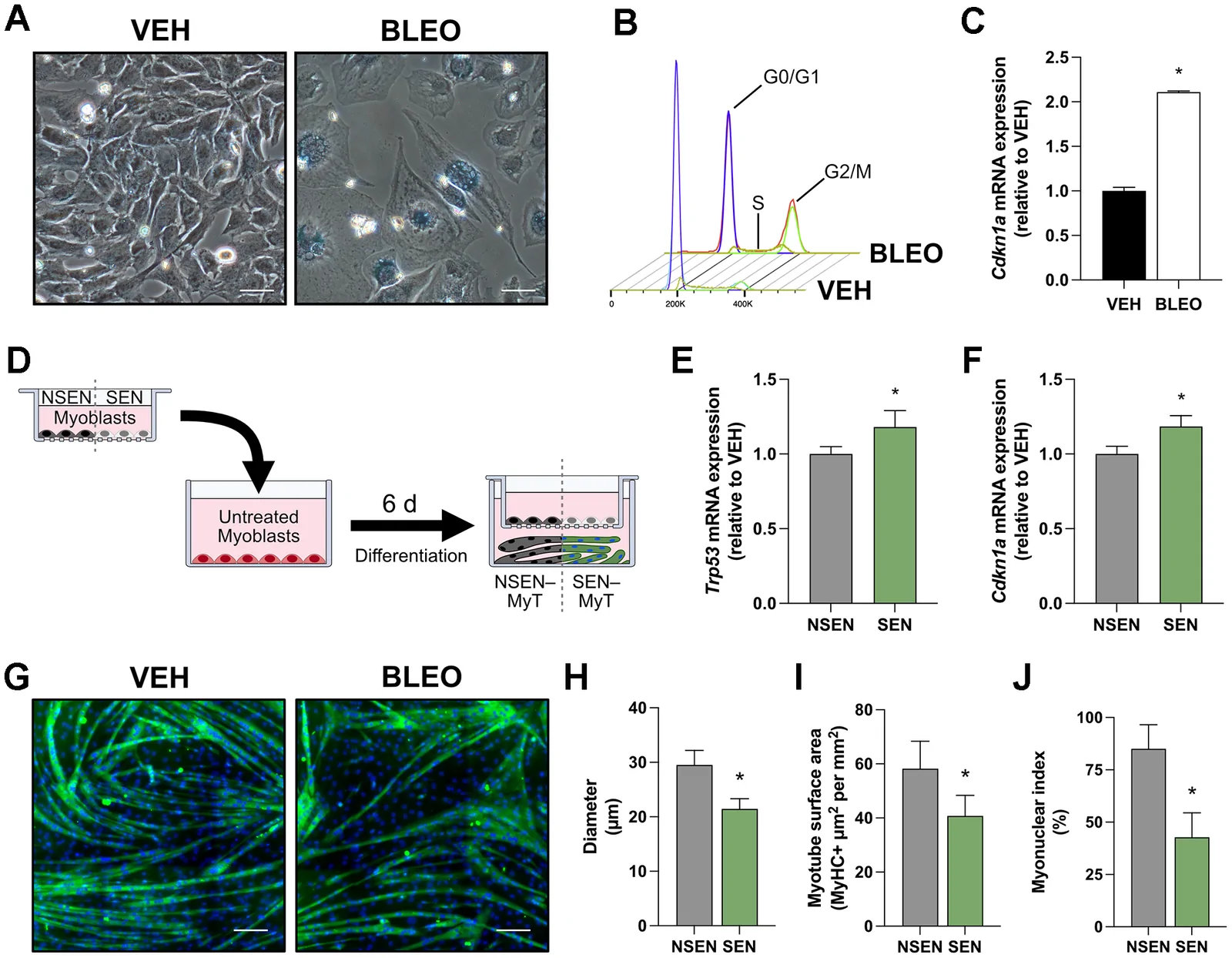
**Transwell culture with senescent myoblasts impairs myotube formation.** (**A**) Representative phase contrast microscopy images of myoblasts stained for SA-β-gal at pH 6.0, captured 48h after dosing with vehicle (VEH) or bleomycin (BLEO). (**B**) Flow cytometry histograms of myoblasts stained with propidium iodide 48h after treatment. The fluorescent intensity of propidium iodide determines the cell cycle phase. (**C**) Relative mRNA expression of *Cdkn1a* (p21) following VEH or BLEO treatment. (**D**) Schematic diagram of myotubes exposed to SEN/NSEN myoblasts in a 0.4-μm transwell insert. This allows for intercellular communication exclusively through factors released into the cell culture media. Relative mRNA expression of senescence genes (**E**) *Trp53* and (**F**) *Cdkn1a* in myotubes after 6 days of 0.4-μm transwell culture with NSEN or SEN myoblasts. (**G**) Representative immunofluorescence microscopy images of myotubes after co-culture with NSEN or SEN myoblasts. Myotubes are stained for MyHC (green) and nuclei (DAPI; blue). Graphical quantification of (**H**) myotube diameter, (**I**) myotube surface area, and (**J**) myonuclear index.

### Significantly altered miRNA expression profile within senescent myoblast-derived EVs

Recent work has implicated extracellular vesicles (EVs) as having a strong effect on C_2_C_12_ myoblast differentiation [18]. Given the pore size in the transwell inserts only permits the exchange of particles smaller than 400 nm, we hypothesized that small EVs are implicated in the observed intercellular signalling. EVs were isolated from the conditioned media (CM) of SEN or NSEN myoblasts after 48h of culturing (Figure 2A). Following isolation, the samples were visually examined through transmission electron microscopy, which morphologically identified the presence of EVs (Figure 2B). Isolates also had positive protein markers of vesicle biogenesis (Alix, TSG101, Syntenin), tetraspanins (CD9, CD63), and vesicle membranes (HSP70) (Figure 2C). The contaminant marker Calreticulin was undetectable via immunoblotting, indicating that samples contained very minimal apoptotic bodies. Particle size evaluation using nanoparticle tracking analysis revealed that the median diameter was ~100 nm, and most particles were within the range of 30 – 200 nm and therefore consistent with expected EV diameters (Figure 2D).

**Figure 2:**
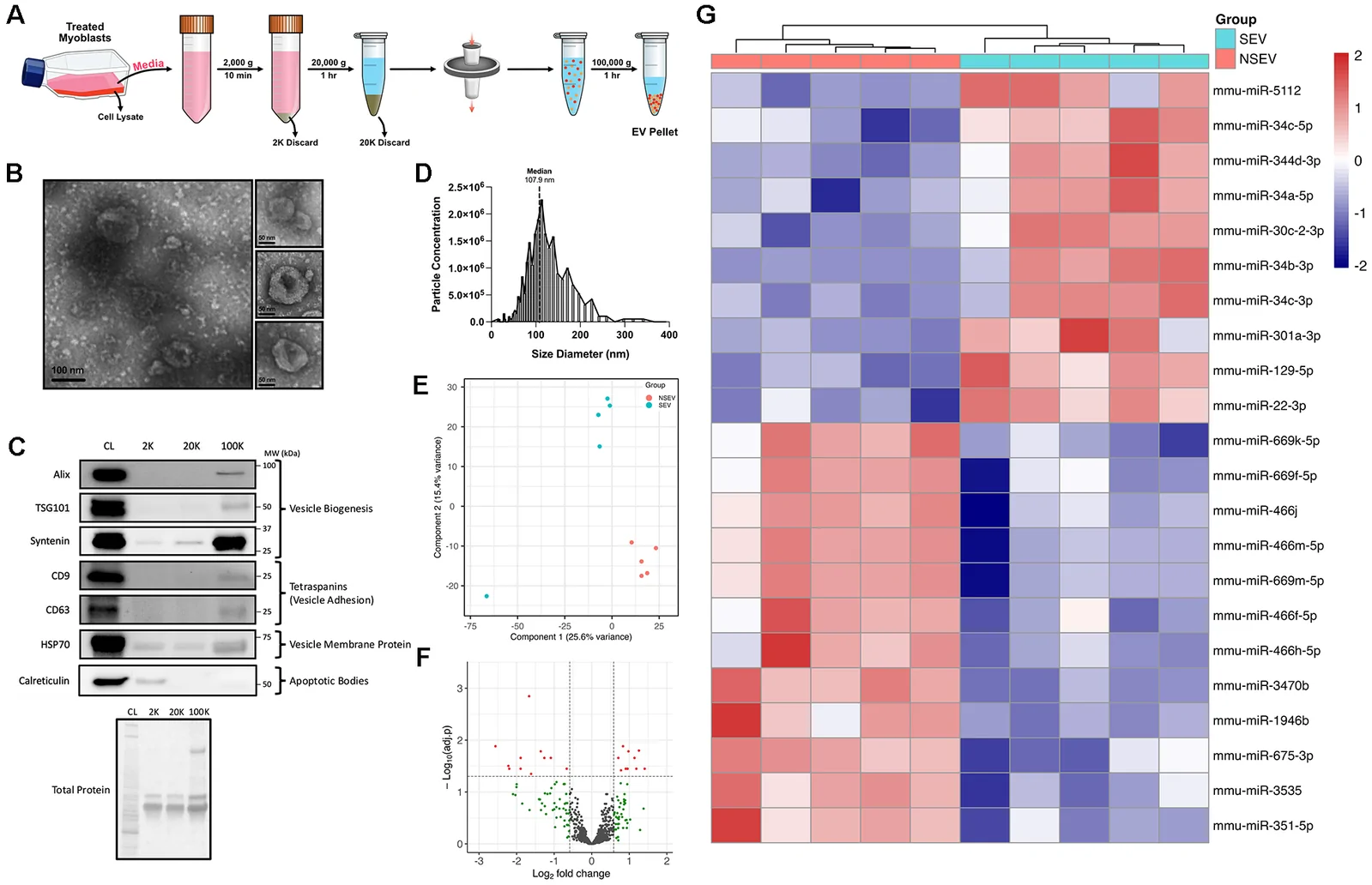
**MiRNA expression profile of EVs released by senescent myoblasts.** (**A**) Schematic diagram of the isolation of extracellular vesicles from the conditioned media of VEH- or BLEO-treated myoblasts. (**B**) Transmission electron micrographs of isolated EVs. Scale bars represent 100 nm and 50 nm. (**C**) Representative western blots of EV-specific markers (Alix, TSG101, Syntenin, CD9, CD63, HSP70) and endoplasmic reticulum contamination markers (Calreticulin) measured in the cell lysate (CL), post-2000g spin discard (2K), post-20,000g spin discard (20K), and the concentrated EV pellet (100K). Blots have been cropped to improve clarity and conciseness of the results. (**D**) EV size and concentration distribution, as measured by nanoparticle tracking analysis. (**E**) Principal component analysis (PCA) of SEN-derived EVs (SEV; cyan) and NSEN-derived EVs (NSEV; orange). (**F**) Volcano plot of differentially expressed (DE) miRNAs based on adj. *p* < 0.05 and FC ≥ |1.5|. Red dots indicate significant DE miRNAs, while green dots are non-significant with FC ≥ |1.5| and adj. *p* > 0.05. (**G**) Heatmap showing all DE miRNA (adj. *p* < 0.05 and FC ≥ |1.5|) with hierarchal clustering of SEV and NSEV samples.

EVs, among other factors, are packaged with small, non-coding RNAs termed microRNAs (miRNAs), which are known to target and post-transcriptionally regulate messenger RNA (mRNA) [14, 15]. Therefore, we assessed the miRNA expression profile of senescent-derived EVs (SEV) and non-senescent-derived EVs (NSEV). Total RNA isolated from EVs was analyzed with a miRNA 4.0 GeneChip array. The principal component analysis of normalized expression data revealed separate clustering of the SEV and NSEV miRNA profiles, with a cumulative variance of 41% across the first two principal components, suggesting distinct genetic profiles between the groups (Figure 2E). We identified 1906 mouse miRNA transcripts, and differential expression analysis (DEA) revealed 22 significant (*adj. p* < 5% and FC ≥ |1.5|) miRNAs (Figure 2F). A heatmap displays the differentially expressed (DE) miRNAs, with 10 upregulated and 12 downregulated in the SEV group relative to NSEV (Figure 2G). To the best of our knowledge, the following miRNAs have not been previously linked to cellular senescence: [upregulated in SEVs] miR-5112, miR-301a-3p; [downregulated in SEVs] miR-669k-5p, miR-669f-5p, miR-466j, miR-466m-5p, miR-669m-5p, miR-466f-5p, miR-466h-5p, miR-3470b, miR-3535, miR-351-5p. Other DE miRNAs have been linked to cell cycle regulation (miR-344d-3p and miR-30c-2-3p) or aging (miR-1946b), but not directly to senescence [19–21]. As miRNAs tend to be evolutionarily conserved across several species, we also performed DEA on all miRNA transcripts present on the array (Supplementary Figure 1). Prior to any statistical comparisons, we filtered out other species’ miRNAs if they had a nucleotide sequence found in any mouse miRNA. We observed that several of the DE mouse miRNAs were also significantly expressed in other species, especially miR-22, which was upregulated across 12 different species. Taken together, these data highlight a distinct miRNA profile in EVs released by senescent myoblasts, with several of these miRNAs being implicated in senescent gene regulation.

### Senescent myoblasts secrete EVs that contain miRNAs implicated in cell cycle regulation, apoptotic signalling, and autophagy

Next, we sought to identify the biological processes of the genes targeted by the DE miRNA. These genes were identified using the miRTarBase, miRecords, and TarBase databases, and only validated targets were included in the analysis. Gene ontology (GO) functional annotation of these genes was conducted, and the biological processes (BP) were clustered into groups using the *clusterProfiler* package in R. A network mapping of the GO BP terms revealed several biological functions associated with cellular senescence, with group themes consisting of cell cycle regulation, autophagy, and apoptotic signalling (Figure 3). Interestingly, there were several BP annotations associated with DNA damage and double-stranded break repair, suggesting that SEVs contain miRNAs that are relevant not only to the senescent condition, but also the stimulus that induced it. Furthermore, SEV miRNAs seem to be implicated in leukocyte regulation, suggesting that they may have SASP-like effects on these immune cells. Taken together, these findings revealed that EVs released by senescent myoblasts are packaged with meaningful miRNA cargo that reflects the current state of the cell and that these SEVs have the potential to be potent SASP members.

**Figure 3:**
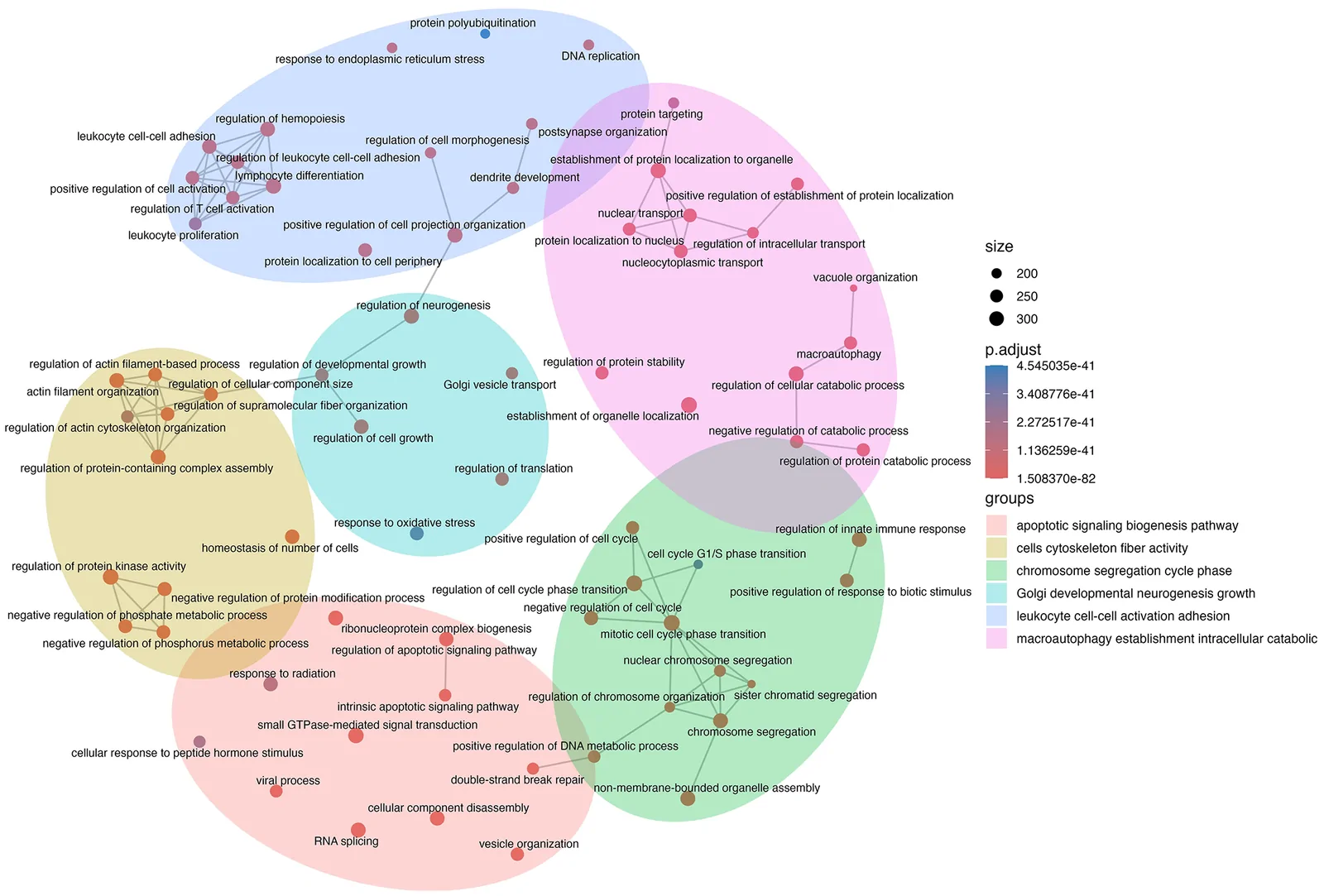
**Network mapping of biological processes implicated by senescent EV miRNAs.** Validated gene targets of differentially expressed miRNAs were analyzed for gene ontology (GO) terms related to biological processes. GO terms were clustered into groups and mapped using the R function *emapplot* in the *enrichplot* package. Node size is proportional to the number of gene targets found in each term, and node colour is related to the adjusted p-value.

We then opted to examine the target genes for the upregulated and downregulated miRNAs separately. Upset plots visually display overlapping gene targets between downregulated miRNAs (Figure 4A) and upregulated miRNAs (Figure 4B). Notably, there were significantly more gene interactions between the upregulated miRNAs than the downregulated miRNAs, most likely due to the identification of more validated gene targets in the former. Several miRNA pairings targeted the same gene sets, such as miR-669m/miR-466m/miR-466j/miR-466h (79 intersections) or miR-34a/miR-301a (1301 intersections). These data imply that a redundancy in gene regulation exists in miRNAs that changed in the same direction within SEVs.

**Figure 4:**
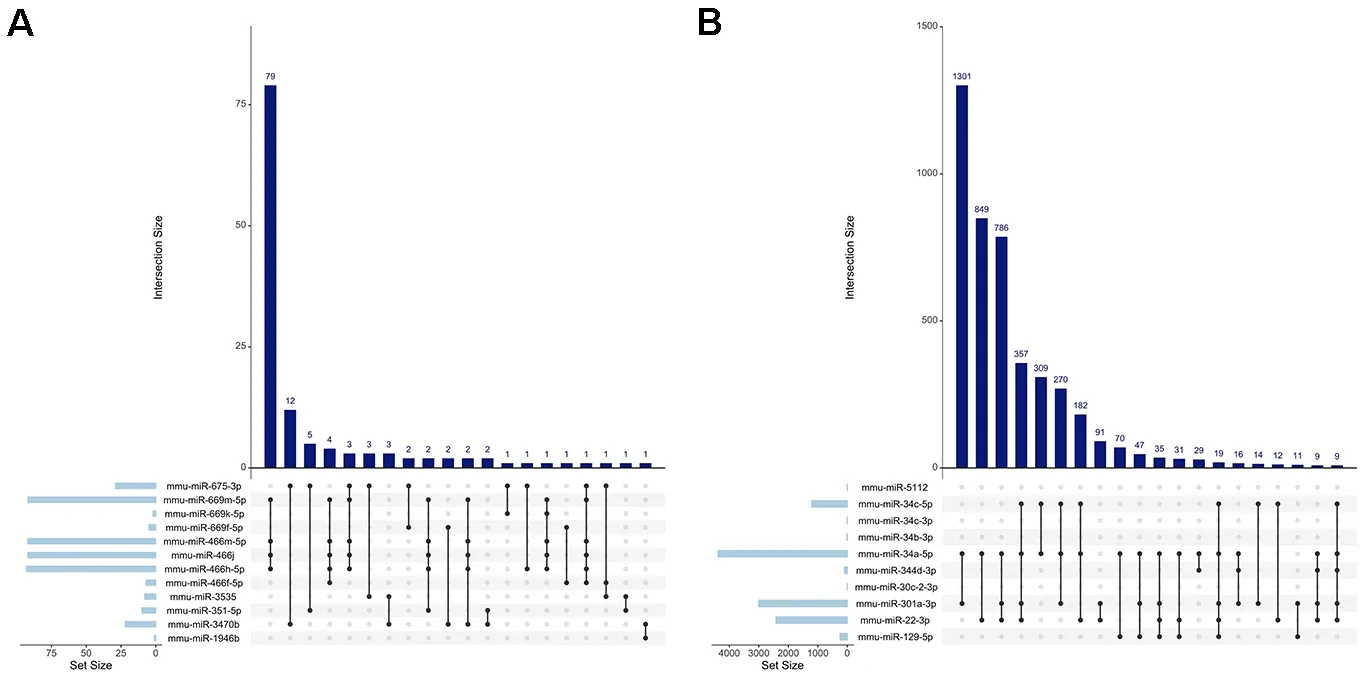
**Visualization of miRNA gene target intersections.** Upset plots depicting the number of shared gene targets between all the (**A**) downregulated miRNAs and (**B**) upregulated miRNAs. The vertical bars display the size of each intersection, while the horizontal bars represent the size of each miRNA set. The connected dots below the vertical bars indicate which sets of miRNAs are included in that intersection.

An interesting finding is the overlap in gene targets between miR-34a-5p, miR-301a-3p, and miR-22-3p (Figure 4B; 786 intersections). While both miR-34a and miR-22 have been extensively associated with cellular senescence, miR-301a has not, suggesting this may be a novel senescence-associated miRNA [22-24]. To examine this further, we mapped out the GO BP term clusters of the three upregulated miRNAs (Supplementary Figure 2). The network revealed significant overlap in biological functions between the genes targeted by miR-34a, miR-301a, and miR-22, with plotted GO terms displaying the proportion of validated genes regulated by each miRNA (pie-chart nodes). Importantly, key biological processes associated with senescence, autophagy, and muscle cell differentiation were all observed. KEGG enrichment analysis revealed that the validated gene targets of miR-301a were the most associated with cell cycle signalling pathways (Supplementary Figure 3), providing further evidence that this miRNA might be implicated in senescent gene signalling.

### Shared regulation of top targeted genes by divergently expressed miRNAs

To identify the genes most likely to be affected by SEV miRNAs, we plotted the interactions between all DE miRNAs and the topmost targeted genes (defined as any gene targeted by more than 5 DE miRNAs; Figure 5). Network visualization was conducted using miRnet.ca, and miRNAs were coloured based on expression (upregulated – red; downregulated – blue), with node size corresponding with the degree of connectivity to displayed target genes. We identified several genes of interest, including *Pten*, which was regulated by the greatest number of DE miRNAs. Another topmost targeted gene of interest was *Mdm2*, a member of the senescence-associated super-enhancer (SASE) family that acts to suppress apoptosis by reducing p53 activity in response to DNA damage [25].

**Figure 5:**
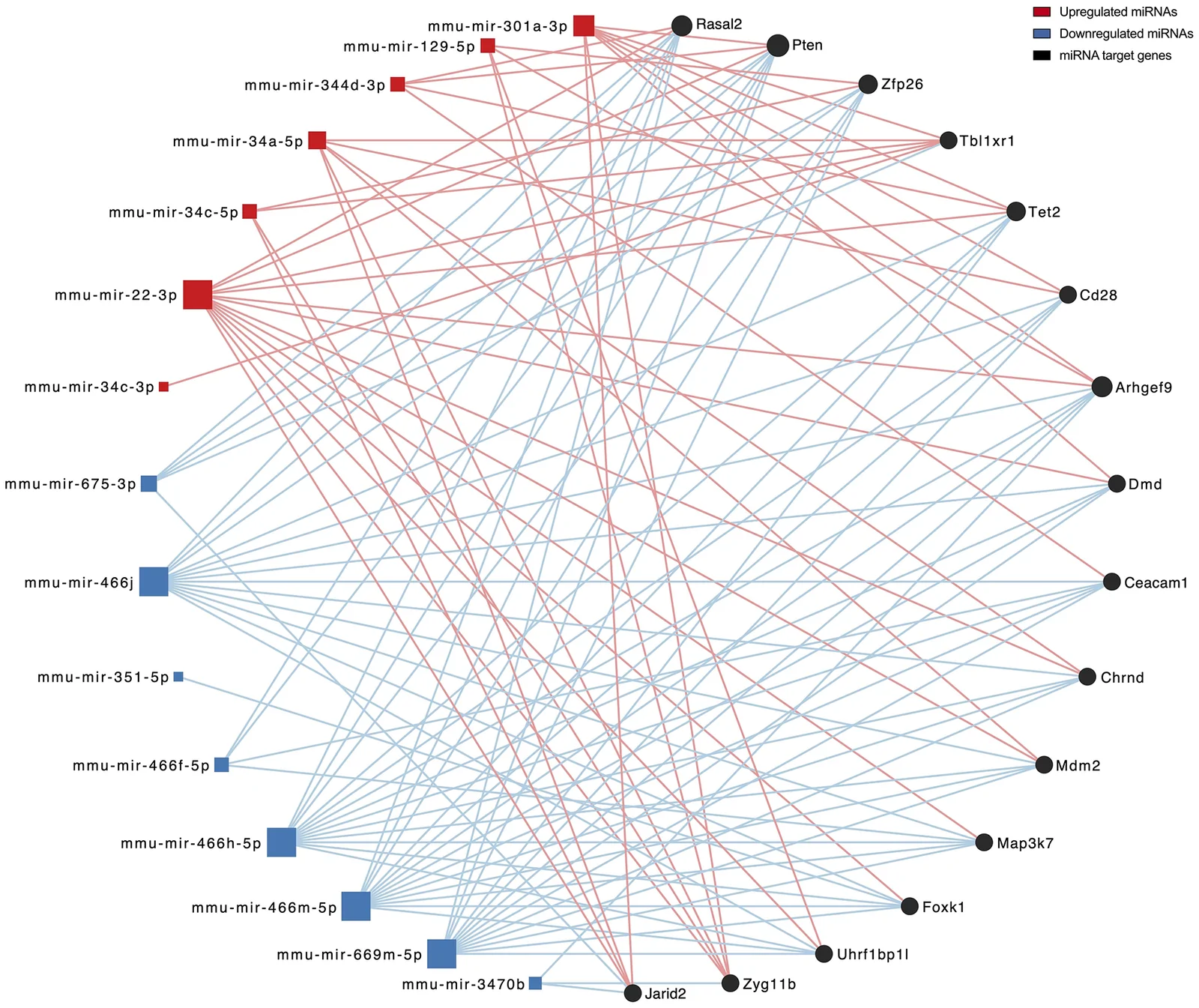
**Network mapping of DE miRNAs and their topmost targeted genes.** A visualization of upregulated (red) and downregulated (blue) miRNAs and the target genes they interact with. Node size is proportional to the degree value of connectivity. The network was degree filtered with a cut-off of 5 to display the top miRNA-targeted genes.

Notably, every gene identified in the network map was regulated by at least one upregulated and one downregulated miRNA. To examine this further, we evaluated the overlap in gene regulation by miRNAs that were divergently changing in SEVs (*i.e.*, how many gene targets do an upregulated and downregulated miRNA share). An Upset plot depicting only the intersections between divergently expressed miRNAs displays the overlaps in gene regulation between upregulated and downregulated miRNAs, with the most intersecting being miR-34a/miR-3470b (62 intersections; Supplementary Figure 4). This finding was unexpectedly high, as the greatest overlap between all downregulated miRNA targets was only 79 intersections (Figure 4A). Altogether these observations support the notion that a change in the expression of a given gene cannot be confidently inferred by examining the directional change of its upstream targeting miRNA.

### Senescent-derived EVs promote anti-apoptotic gene signalling

Given the potential for SEV-mediated regulation of gene signalling, we next sought to experimentally determine if EV treatment can affect the genetic expression of recipient cells. Isolated NSEVs and SEVs were added to C_2_C_12_ myoblasts for a total of 48h, after which the cells were washed and collected for RT-qPCR (Figure 6A). There was no effect of SEV transfer (SEV–T) on senescence markers *Trp53* (Figure 6B) or *Cdkn1a* (Figure 6C) relative to NSEV transfer (NSEV–T). However, the expression of two of the most targeted genes by DE miRNAs did change; an elevation in *Pten* (+52%; *p* < 0.05; Figure 6D) and *Mdm2* (+56%; *p* < 0.05; Figure 6E) mRNA was observed in myoblasts co-incubated with SEVs. Interestingly, SASP-related genes *Il33* and *Cyr61* decreased with SEV–T (−52%, −26%; *p* < 0.05; Figure 6F–6G). These findings demonstrate that SEVs released from myoblasts are capable of regulating genes targeted by their packaged miRNAs. Furthermore, SEVs do not elicit a senescent genotype but rather promote anti-apoptotic and anti-SASP signalling.

**Figure 6:**
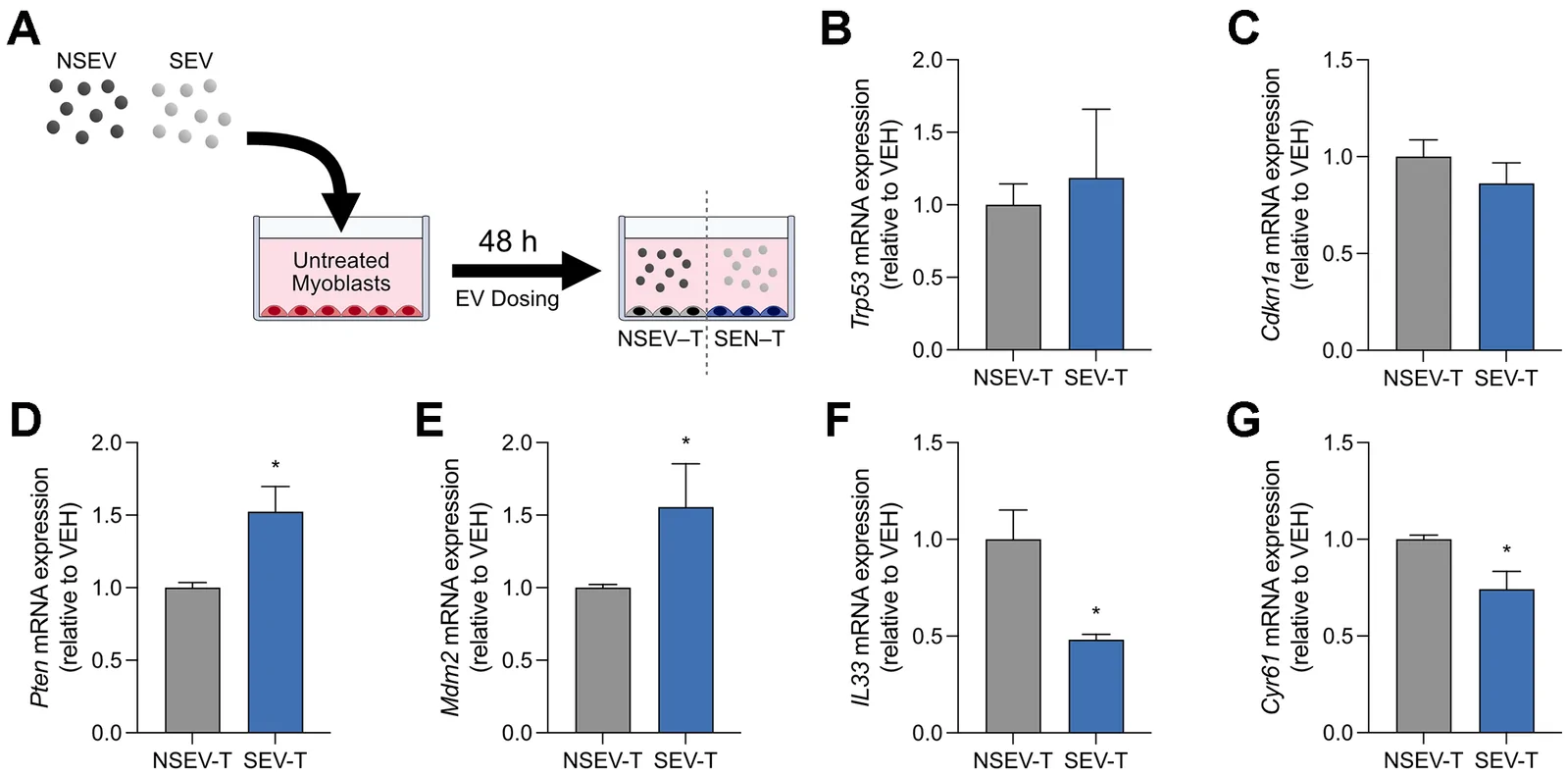
**Co-culture transfer experiments of senescent-derived EVs onto myoblasts.** (**A**) Schematic diagram of myoblasts cultured with EVs derived from senescent (SEV) or non-senescent (NSEV) myoblasts. Relative mRNA expression of senescence genes (**B**) *Trp53* and (**C**) *Cdkn1a* within myoblasts 48h following the transfer of NSEV or SEV. Relative mRNA expression of top DE miRNA gene targets (**D**) *Pten* and (**E**) *Mdm2*. Relative mRNA expression of SASP genes (**F**) *Il33* and (**G**) *Cyr61*. Data are means ± SD. * denotes a significant difference from the NSEV-T group (*p*<0.05). SEV-T: Senescent EV-treated myoblasts; NSEV-T: Non-senescent EV-treated myoblasts.

### SEV miRNA gene targets are differentially expressed in aged mouse muscle

Lastly, we sought to investigate whether senescent EV-containing miRNAs act on genes that are altered in aging skeletal muscle. RNA-Seq data from the gastrocnemius muscle of adult (8-month) and geriatric (28-month) C57BL/6JRj mice were obtained from previous work [26]. Börsch and colleagues (2021) identified 2302 genes that were differentially expressed (FDR < 5% and FC ≥ |1.5|) in aged skeletal muscle (Figure 7A). Of these, 700 differentially expressed genes (DEG) are also validated gene targets of 2 or more SEV DE miRNAs as found in our dataset, representing a significant overlap (*p* = 4.52 × 10^−14^). Together, these observations imply that a meaningful relationship exists between the aging-related genetic alterations in skeletal muscle and the miRNAs released by senescent cells. We next performed KEGG enrichment analysis on the 700 overlapping DEG and identified the top 10 significant pathways (Figure 7B). Notable amongst these were pathways related to cellular senescence, autophagy, and FoxO signalling. Heatmaps display the significant DEG (between 8mo and 28mo gastrocnemius muscle) implicated in cellular senescence and apoptosis, with only the genes targeted by two or more SEV miRNAs being displayed (Figure 7C). Despite only 14.6% (700/4800) of SEV miRNA targets being altered in muscle during aging, these genes are heavily implicated in pathways related to MuSC function, muscle differentiation, autophagy, apoptosis, and cellular senescence [6, 27, 28].

**Figure 7:**
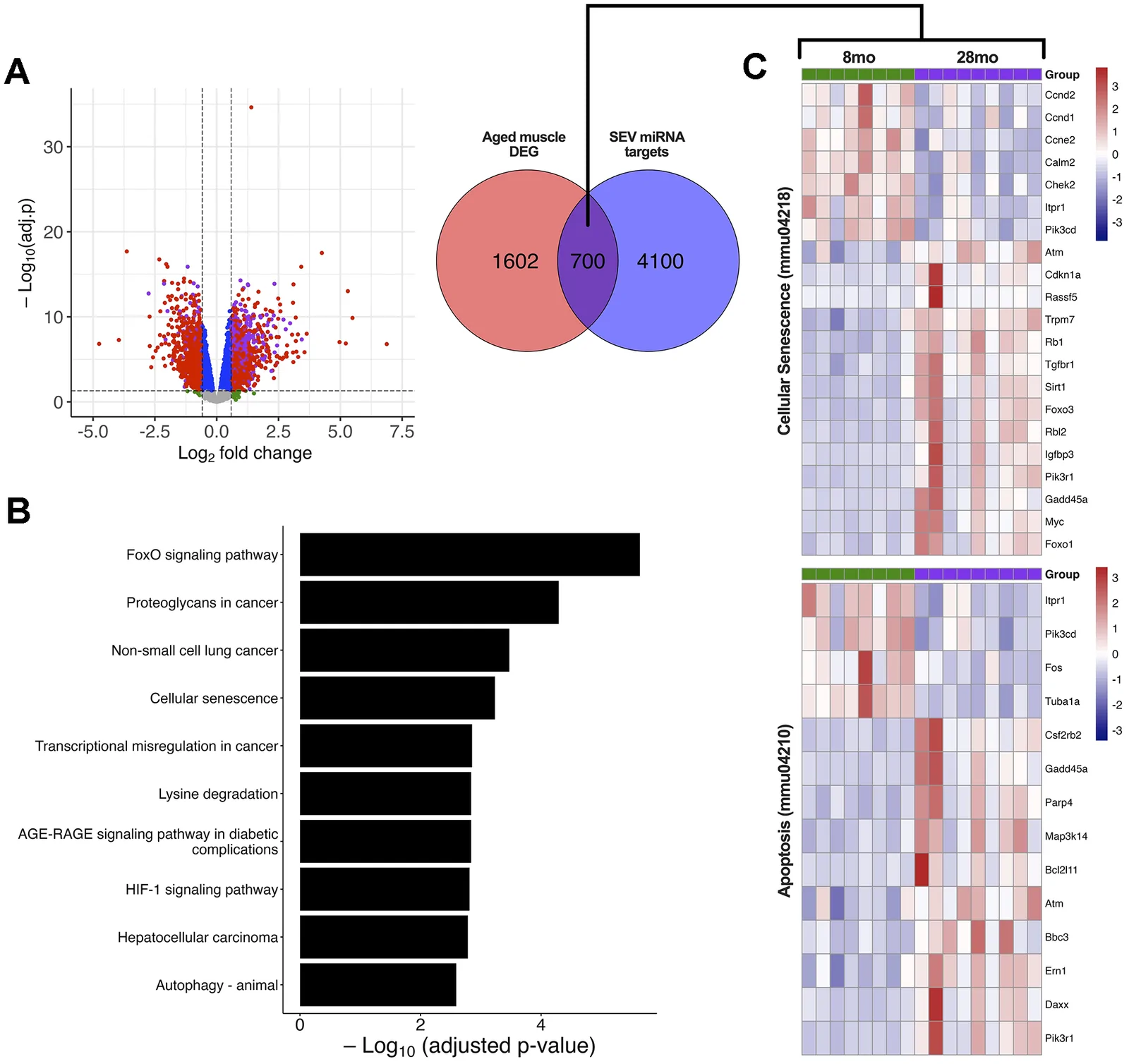
**Association of SEV-derived miRNAs with the transcriptomic profile of geriatric mouse muscle.** RNA-seq data of gastrocnemius muscle from young (8mo; n = 8) and geriatric (28mo; n = 9) C57BL mice. (**A**) Volcano plot of differentially expressed genes (DEG) based on adj. *p* < 0.05 and FC ≥ |1.5|. Red dots indicate significant DEG, while purple dots are significant DEG that are validated gene targets of SEV miRNA. Green dots are non-significant with FC ≥ |1.5| and adj. *p* > 0.05. A Venn diagram illustrates the overlap (purple) between aged gastrocnemius DEG and genes targeted by two or more DE miRNAs within SEV. (**B**) KEGG enrichment analysis of overlapping geriatric DEG and SEV miRNA targets. The top 10 significant pathways are plotted in descending order by adj. *p*-value. (**C**) Heatmap showing gene expression data from 8mo and 28mo gastrocnemius muscle for two significant KEGG pathways, cellular senescence (mmu04218) and apoptosis (mmu04210). All genes displayed are significant (FDR < 0.05 and FC ≥ |1.5|) and validated targets of 2 or more SEV miRNA. RNA-seq data was obtained from publicly available data (GSE145480) [26].

## DISCUSSION

Cellular senescence is a hallmark of aging that has been implicated in the dysfunction of satellite cells in aged skeletal muscle [5, 29]. In this study, we investigated the potential of extracellular vesicles, released from senescent cells, to play a role in propagating the SASP. We first observed that senescent myoblasts secrete factors that impaired the differentiation of co-incubated myotubes. Given the possibility that EVs could mediate this intercellular communication, we analyzed their miRNA expression profiles and determined that senescent myoblast-derived EVs (SEVs) carry cargo containing miRNAs that regulate cell cycle and DNA damage signalling. Furthermore, miR-301a-3p was identified as a novel senescence-associated miRNA. Despite the prominent targeting of senescent gene signalling by SEV-miRNAs, SEV transfer onto myoblasts does not appear to promote cellular senescence. Rather, SEVs increased anti-apoptotic gene expression in recipient myoblasts. Lastly, we identified that a substantial proportion of SEV miRNA gene targets are differentially expressed within the skeletal muscles of geriatric mice and that these genes are significantly implicated in senescent signalling pathways. Overall, the results of this study suggest that the miRNA cargo of EVs has the potential to regulate senescent gene expression and may contribute to some of the aging-related perturbations in the muscle transcriptome.

In the present study, when healthy myoblasts were co-cultured with senescent cells over a 6-day period, the exchange of factors through the media significantly impaired myoblast differentiation, supporting the notion that senescent myoblasts are capable of negatively affecting muscle through extracellular communication. Given that multiple investigations have identified the miRNA cargo of EVs as the driving force behind intercellular senescence-associated signalling [30–32], we hypothesized that small EVs may play a role in the impairment of myotube formation following senescent co-culture. In the present study, microarray profiling of SEVs released by senescent myoblasts identified 22 DE mouse miRNAs. Network clustering of GO terms showed that these genes are significantly implicated in biological processes related to cell cycle regulation, DNA damage repair, apoptosis, autophagy, and vesicle organization. Previous work has found that EVs released by myoblasts or myotubes contain proteins and miRNAs that are related to vesicle biology rather than the conditions of the origin cells [33, 34]. In contrast, we have shown that EVs from senescent myoblasts are packaged with miRNAs that reflect the status of their host cell. Several notable senescence-associated miRNAs were elevated in SEVs relative to non-senescent-derived EVs (NSEVs), including miR-34a, -34b, -34c, and -22. The overexpression of miR-34a/b/c or miR-22 has been linked to senescence induction in multiple studies, including within lung epithelial cells, human fibroblasts, and multiple cancer cell lines, primarily by acting on genes involved with tumour suppression [22–24, 35]. Furthermore, prior research has revealed that aging mice have elevated levels of miR-34a in circulating muscle-derived EVs [16].

MiR-301a stands out as a potential senescence regulator on par with miR-34a and miR-22. Presently, the majority of the literature available surrounding miR-301a is related to its use as a diagnostic or prognostic biomarker for several types of cancer, including prostate, breast, brain, and colorectal [36–39]. However, there are several lines of evidence that suggest this miRNA could be a prominent senescence mediator. Through the examination of intersecting (overlapping) target genes, we identified that miR-301a-3p shares 786 gene targets with miR-34a-5p and miR-22-3p. Pathway analysis of miR-301-regulated genes revealed that the most significant process implicated was cell cycle regulation. Furthermore, a GO cluster network showed that the top biological pathways that overlap between the gene targets of miR-301a, miR-34a, and miR-22 were related to cell cycle phase transition, autophagy regulation, and mRNA translation. MiR-301a is capable of stimulating NF-κB activity, which we and others have shown acts to increase levels of the senescence gene *Cdkn1a* (p21) [17, 40, 41]. Furthermore, lentiviral-mediated overexpression of miR-301a increased the production of prominent SASP factor TNF-α by CD4+ T cells [42]. There also exists a regulatory relationship between miR-301a and one of its target genes, phosphatase and tensin homolog (PTEN). PTEN is a major negative regulator of the PI3K/AKT signalling pathway, and its loss triggers senescence through p53-dependent mechanisms [43, 44]. Interestingly, the existing literature is counterintuitive with respect to the change in PTEN and cell proliferation status following miR-301a overexpression. Some papers reported that elevated miR-301a decreases PTEN, which subsequently improves cell proliferation [45–47]. Jung et al. suggested that one possible explanation for this contradictory increase in proliferation revolves around the activity of the mechanistic target of rapamycin (mTOR) [48]. In their paper, they proposed that when proliferation signals are consistently triggered by the loss of PTEN, mTOR directly phosphorylates p53 to induce premature senescence as a protective mechanism against tumorigenesis [48]. Applying this logic to the present work suggests that continuous exposure to SEVs for an extended period has the potential to induce senescence through a miR-301a–PTEN–mTOR signalling axis. These conclusions would also explain why a 48-h treatment with SEVs did not elicit a senescent phenotype.

To evaluate the regulatory potential of SEV miRNAs, we treated myoblasts with EVs isolated from senescent/non-senescent cells before measuring the genetic expression of several highly regulated SEV miRNA gene targets. We chose to examine *Pten* mRNA because it was the most common targeted gene amongst all DE miRNA, and because of its association with cellular senescence [44, 48]. *Mdm2* was also one of the most targeted by DE miRNA and is a known member of the SASE family of super-enhancer genes [25]. Both *Pten* and *Mdm2* expression increased in myoblasts following co-incubation with SEVs, thereby confirming that EVs can regulate the genetic targets of their miRNA cargo. SASE genes, such as *Mdm2*, act to promote cell survival during periods of cellular stress by repressing p53-dependent apoptosis while allowing the NF-κB-mediated activation of p21 and subsequent induction of cellular senescence [17, 49]. Crucially, mTOR complex 1 (mTORC1) and mTORC2 compete with MDM2 for the regulation of p53 [48]. MDM2 typically binds to p53, but when PTEN levels are diminished, both mTOR complexes are activated and preferentially phosphorylate p53 to trigger cellular senescence [48, 50]. In the current work, the observed upregulation of *Pten* and *Mdm2* in SEV-treated myoblasts is consistent with anti-apoptotic signalling observed in the early stages of senescence induction [17]. Moreover, the observations in the current study align with previous research showing that EVs derived from senescent human dermal fibroblasts exert anti-apoptotic effects through their miRNA contents [30]. Therefore, we suggest that senescent myoblasts are secreting SEVs in an attempt to “prime” neighbouring cells towards the incoming SASP stimulus and prevent premature cell death before senescence can be fully initiated. We also propose *Pten* as a potential member of the SASE family of apoptosis regulators.

Myoblasts treated with SEVs did not change the expression of *Cdkn1a* or *Trp53,* suggesting that EVs secreted in the early stages of senescence induction are not capable of rapidly inducing senescent signalling. This finding is consistent with prior studies that showed that senescent-derived EVs increased cell proliferation and promoted fibrosis rather than inducing senescence [32]. It is possible that any potential upregulation of *Trp53* by SEVs was counteracted by the increase in *Mdm2* expression. Bleomycin treatment in these cells has been shown to induce senescence via p21 activation independently of p53, so there is potential that this gene’s activity is being diminished by opposing upstream regulators [17]. While there was no activation of senescent genes following SEV co-incubation, it is feasible that this could change with longer treatment durations or by using EVs isolated from cells with a more prominent senescent phenotype. Alternatively, other released SASP factors may drive senescence induction, with EVs primarily acting as supporting contributors. While these SEV co-incubation experiments primarily focused on transcriptional responses in proliferating myoblasts, future studies assessing their effects on nascent myotubes would help determine whether senescent-derived EVs similarly influence myogenic differentiation.

The skeletal muscle transcriptome changes significantly throughout the lifespan of both mice and humans [26]. Integrating the microRNAome analyses from our study with RNA-seq data from young and aged mouse gastrocnemius muscle demonstrated that there was a significant overlap between senescent targets and genes altered during aging. Even after filtering out genes that were only regulated by one DE miRNA, we identified 700 DEG in geriatric muscle that are validated gene targets of SEV miRNAs. Importantly, these 700 genes are implicated in pathways related to sarcopenia, including cellular senescence, FoxO signalling, and autophagy [6, 27, 28]. Several genes implicated in apoptosis signalling were also significantly DE in aged muscle, which supports the observations in SEV-treated myoblasts. Taken together, the findings discussed here indicate that a sizable proportion of the genes altered in aged muscle can be regulated by senescent EVs, suggesting that these small particles could play a role in the onset, progression, or maintenance of sarcopenia.

The present study does have limitations worth addressing. The focus of this work was on the miRNA cargo of senescent myoblast-derived EVs, but these small particles are known to carry other components such as proteins, lipids, and other RNAs, which could also contribute to the biological effects observed here. Additionally, the EV isolates presumably contained a heterogeneous vesicle population, including exosomes and microvesicles, although the NTA results showed an enrichment of small (< 200 nm) particles, indicating that the majority of the samples were likely small EVs. However, it is possible that the isolates contained other populations, such as lipoproteins or protein complexes, that are known to carry miRNAs and are capable of eliciting a senescent response in an EV-independent manner [51–54]. Therefore, we cannot definitely state that small EVs were primarily responsible for the findings reported here. Lastly, as with all *in vitro* studies, caution must be exercised when extrapolating findings to *in vivo* muscle tissues, especially given the use of C_2_C_12_ myoblasts in the present study.

In conclusion, senescent myoblasts produce factors that impair the differentiation of neighbouring myotubes. Small extracellular vesicles released from these cells are promising candidates for this phenomenon as they are packaged with miRNA specific to the cellular conditions of the originating source. Several of these, including miR-34a and miR-22, are heavily implicated in senescence induction, while others, such as miR-301a, have been identified as novel senescence-associated miRNAs. The transfer of senescent-derived EVs onto myoblasts upregulates anti-apoptotic signalling, most likely to prevent premature cell death prior to the induction of senescence by other secreted SASP factors. Lastly, the miRNAs packaged within these EVs target a substantial subset of the genes that change in aged skeletal muscle, suggesting a potential link between these small particles and the regulation of the sarcopenic transcriptome.

## MATERIALS AND METHODS

### Cell culture

C_2_C_12_ myoblasts (American Type Culture Collection) were cultured in a humidified, 5% CO_2_ incubator at 37° C with growth medium (GM) composed of Dulbecco’s Modified Eagle’s Medium (DMEM) supplemented with 10% fetal bovine serum (FBS) and 1% penicillin/streptomycin (Invitrogen). Bleomycin sulfate (Cat. #13877; Cayman Chemical) was utilized to induce senescence as established in previous work from our group [17]. Briefly, bleomycin was dissolved in nitrogen-purged dimethyl sulfoxide (DMSO; Thermo Fisher Scientific) and diluted to 14 μM in GM. At ~50-60% confluence, C_2_C_12_ myoblasts were treated with bleomycin (BLEO) or vehicle (VEH; isovolumetric nitrogen-purged DMSO) for 12h. Cells were then washed twice with sterile 1X phosphate-buffered saline (PBS; Gibco) and replaced with fresh GM or differentiation media (DM), based on experimental design. A senescent phenotype was confirmed in this model using senescence-associated β-galactosidase staining, cell cycle flow cytometry, *Cdkn1a* mRNA expression and γH2AX immunocytochemistry [17]. Differentiation was induced with differentiation medium composed of DMEM supplemented with 2% horse serum (Invitrogen) and 1% penicillin/streptomycin; DM was changed every 48h. Experiments were conducted with cells at passages 6-8, and technical duplicates were averaged to create each *N*, with a total *N* = 3 per condition. The C_2_C_12_ cell line was authenticated prior to use, and cells were routinely examined for mycoplasma contamination throughout the study.

### Senescence-Associated β-galactosidase activity

To histologically identify senescent myoblasts, treated cells were stained for Senescence-Associated β-galactosidase (SA-β-gal) 48h after treatment. Briefly, the myoblasts were fixed with 1X fixative solution (Cell Signalling Technologies) for 10 min. After washing with PBS, β-galactosidase staining solution (pH 6.0, Cell Signalling Technologies) was added, and the plates were stored in a dry, 37° C incubator for 16h. Images were captured through phase-contrast microscopy on the Eclipse TI2 microscope (Nikon), 20X objective, and analyzed with NIS-Elements (V5.30.01, Nikon).

### Flow cytometry

Myoblasts were treated with BLEO or VEH for 12h, then cultured in GM for 48h before being washed twice, collected, and filtered through a 35-μm cell strainer into flow tubes (Corning). Cells were then pelleted, and ice-cold 70% ethanol was added dropwise under gentle agitation. Myoblasts were fixed for 30 min at 4° C and then washed twice with 1 × PBS before being resuspended in 500 μL of propidium iodide (PI) solution (5 μg/mL of PI and 20 μg/mL of RNase A in 1×PBS). Cell cycle kinetics were determined based on PI fluorescence using a Cytoflex LX (Beckman Coulter) and quantified with FlowJo (V10.8.1, BD Biosciences). Staining dyes used for flow cytometry are detailed in Table 1.

**Table 1:** Antibody and dye information.

**Western blotting**					
**Antibody**	**Host**	**Clone**	**Dilution^a^**	**Manufacturer**	**Cat. #**
Anti-Alix	Mouse	3A9	1:1000	CST	2171S
Anti-TSG101	Rabbit	EPR7130	1:1000	Abcam	ab125011
Anti-Syntenin	Rabbit	-	1:1000	Abcam	ab19903
Anti-CD9	Rabbit	EPR23105-125	1:1000	Abcam	ab223052
Anti-CD63	Rabbit	EPR5702	1:1000	Abcam	ab216130
Anti-HSP70	Rabbit	EPR181606	1:1000	Abcam	ab181606
Anti-Calreticulin	Rabbit	D3E6	1:2000	CST	12238S
**Immunocytochemistry**					
**Antibody/Dye**	**Host**	**Clone**	**Concentration**	**Manufacturer**	**Cat. #**
Anti-MyHC I	Mouse	A4.951	Neat	DSHB	A4.951
DAPI	–	–	0.33 μg/mL	Sigma-Aldrich	D9542
**Flow Cytometry**					
**Dye**	**Host**	**Clone**	**Concentration**	**Manufacturer**	**Cat. #**
Propidium Iodide	–	–	5 μg/mL PBS	Invitrogen	P1304MP

^a^ WB dilutions were in 5% BSA in TBS-T.

### Transwell co-culture

Myoblasts were seeded onto a transwell insert with a 0.4-μm membrane pore (Corning, Falcon) and treated with BLEO or VEH (as stated above) to generate senescent (SEN) or non-senescent (NSEN) myoblasts. The insert was placed into a well that was seeded with untreated myoblasts 12h earlier, which allowed for intercellular communication between SEN or NSEN myoblasts and the untreated cells, without permitting direct cell-cell interactions. Untreated myoblasts were then allowed to proliferate to ~90% confluence before changing to a differentiation medium (DM) composed of DMEM supplemented with 2% horse serum (Invitrogen) and 1% penicillin/streptomycin. Every 48h, DM was changed and the inserts were replaced to prevent over-confluence. Differentiation was induced after 6 days of culturing with DM, and myotubes were collected for ICC or RT-qPCR.

### RNA isolation and real-time quantitative PCR (RT-qPCR)

Myoblasts or myotubes were collected with Trizol reagent (Invitrogen), and RNA was purified using the E.Z.N.A. Total RNA Kit (Omega Bio-Tek) as per manufacturer instructions. Isolated RNA concentration and purity were measured with the Nanodrop 1000 spectrophotometer (Thermo Fisher Scientific), after which 1 μg of RNA was reverse transcribed into cDNA using a high-capacity cDNA reverse transcription kit (Thermo Fisher Scientific). Gene amplification was performed using TaqMan assays (Table 2) and Taqman Fast Advanced Master Mix (Thermo Fisher Scientific). Cq values were obtained in triplicate and relative gene expression data were normalized to the geometric mean of reference genes *Gapdh* and *Tbp* using the comparative 2^-ΔΔCq^ method [55]. A no-template control (NTC) was used for each probe to confirm the absence of contamination or non-specific amplification.

**Table 2:** TaqMan gene probe information.

**Gene**	**Gene name**	**Assay ID^a^**
*Trp53*	Transformation related protein 53 (p53)	Mm01731287_m1
*Cdkn1a*	Cyclin-dependent kinase inhibitor 1A (p21)	Mm00432448_m1
*Pten*	Phosphatase and tensin homolog	Mm00477208_m1
*Mdm2*	Mouse double minute 2	Mm01233138_m1
*Cyr61*	Cysteine rich protein 6	Mm00487498_m1
*Il33*	Interleukin 33	Mm00505403_m1
*Gapdh*	Glyceraldehyde-3-phosphate dehydrogenase	Mm99999915_g1
*Tbp*	TATA box binding protein	Mm01277042_m1

^a^ Assays were manufactured by Thermo Fisher SCientific.

### Immunocytochemistry

Differentiated myotubes were washed with 1X PBS and fixed with 4% paraformaldehyde (PFA) for 10 min. Myotubes were permeabilized for 20 min with 0.1% Triton-X in PBS before blocking with 2% bovine serum albumin (BSA) in PBS for 1h. Myosin heavy chain (MyHC) supernatant was incubated overnight at 4° C (Table 1). Myotubes were then washed twice with 1X PBS and incubated with Alexa Fluor^®^ 488 goat anti-mouse secondary antibody (Invitrogen) at 1:500 in PBS for 2h at room temperature. After two washes, DAPI (4’,6-diamidino-2-phenylindole; Sigma-Aldrich) was added for 5 min to label nuclei. Myotubes were imaged at 20X magnification using an Eclipse TI2 microscope (Nikon) and analyzed with NIS-Elements (V5.30.01, Nikon). Images were quantified to assess myotube diameter (μm), myotube surface area (MyHC+ μm^2^ per image mm^2^), and myonuclear index (%).

### Extracellular vesicle isolation

Extracellular vesicles (EVs) were isolated from the CM of senescent myoblasts in line with the MISEV recommendations for cell culture-conditioned medium [9]. Myoblasts were treated with VEH or BLEO for 12h and then washed twice with PBS. Cells were then incubated for 48h with DMEM supplemented with 10% exosome-depleted FBS (Cat. #A2720801; ThermoFisher Scientific) and 1% penicillin/streptomycin (GM-EV). 5 mL of conditioned media, controlled between experimental conditions, was collected and centrifuged at 2000 *g* for 10 min to pellet cell debris. Samples underwent another centrifugation at 20,000 *g* for 1h at 4° C to remove larger EVs (*i.e.*, microvesicles and apoptotic bodies). The supernatant was collected and filtered through a 0.4-μm pore before being concentrated by ultracentrifugation at 100,000 *g* for 16h at 4° C. EV concentrates were washed twice with 1X PBS prior to final suspension. Ultracentrifugation was performed with the Optima^TM^ MAX-XP (Beckman Coulter) in appropriate polypropylene microtubes (Cat. #357448; Beckman Coulter) and the presence of EVs was confirmed with western blotting, transmission electron microscopy, and nanoparticle tracking analysis as previously described [56]. In accordance with MISEV2023 guidelines, the general term “EV” will be used throughout this manuscript in place of more common terms such as exosome or exosome-like vesicles [9].

### Protein extraction and immunoblotting

Isolated EVs were lysed with ice-cold RIPA buffer (Sigma-Aldrich) supplemented with Pierce protease inhibitor (Thermo Fisher Scientific) and phosphatase inhibitor (Sigma-Aldrich). Samples were sonicated and then centrifuged at 14,000 *g* for 15 min to pellet debris. Protein concentrations were determined using a bicinchoninic acid assay (BCA; Thermo Fisher Scientific), and samples were prepared in 4X Laemmli Buffer (Bio-Rad). Samples were then loaded into each lane of a 4 – 15% TGX precast gel (Bio-Rad) and underwent SDS-PAGE. Following electrophoresis, proteins were transferred to a nitrocellulose membrane and stained with Ponceau S solution (Sigma-Aldrich) to assess equal loading between samples. Ponceau solution was removed with 3 x 5-min washes with Tris-buffered saline with 0.1% Tween-20 (TBS-T), and membranes were blocked with 5% BSA in TBS-T for 1h. Membranes were then incubated overnight at 4° C with primary antibodies diluted in 5% BSA in TBS-T (Table 1). The next day, membranes were again washed with TBS-T and incubated with the appropriate HRP-conjugated secondary antibody (Thermo Fisher Scientific) diluted at 1:10000 in 5% BSA in TBS-T for 2h. Bands were visualized upon the addition of ECL solution (Bio-Rad). Images were captured with the ChemiDoc MP Imaging System (Bio-Rad) and quantified using Image Lab software (V6.0.1, Bio-Rad).

### Transmission electron microscopy

Following EV isolation and concentration, EV pellets were diluted 1:20 into nuclease-free water and 5 μL was added to a Cu/Pd grid (200 mesh with Formvar). Afterwards, grids were air-dried for 10 min then gently dried with filter paper before being stained with 1% uranyl acetate for 1 min. Samples were visualized with a JEOL JEM 1200EX TEMSCAN transmission electron microscope with an HV of 80kV. The presence of EVs was verified with images taken at 40,000X – 150,000X magnification.

### Nanoparticle tracking analysis

Isolated EV samples were diluted 1:500 in ultrapure water (DNase/RNase-free and 0.1-μm membrane-filtered; Invitrogen) and analyzed for size and concentration, as detailed previously [56]. Before and after each sample was measured, approximately 5 mL of ultrapure water was flushed through the cell. Diluted EV samples were injected, and particle size and concentration were measured at 11 different positions, with three readings per position. Pre-acquisition parameters: temperature – 23° C; camera sensitivity – 85; shutter time – 120; and frame rate – 30 fps. Post-acquisition parameters: minimum brightness – 30; minimum size – 5 pixels; and maximum size – 200 pixels. Samples were analyzed with a ZetaView PMX 110 (Particle Metrix).

### miRNA microarray

Total RNA was extracted from EVs isolated from 10 ml of CM for each sample. Two technical replicates were combined (5 mL CM each) to produce each sample, and after ultracentrifugation, 50 μL aliquots of purified EVs were stored at -80° C until processed for RNA isolation. To promote the recovery of small RNAs, the MagMAX *mir*Vana Total RNA Isolation Kit (Cat. #A27828; Applied Biosystems) was utilized in conjunction with the KingFisher Apex purification system (ThermoFisher Scientific). RNA concentration and purity were measured using the Qubit 4 fluorometer (Invitrogen) and Nanodrop spectrophotometer (Thermo Fisher Scientific). Total RNA was then labelled using FlashTag Biotin HSR for miRNA microarrays (Applied Biosystems) to prepare samples for hybridization.

Biotin-labelled samples were combined with a hybridization master mix and incubated at 99° C for 5 min, then 45° C for 5 min. Sample cocktail was then injected into a miRNA 4.0 GeneChip cartridge array (Affymetrix) and placed into hybridization ovens to incubate at 48° C and 60 rpm for 18h. Arrays were then washed with a GeneChip Fluidics Station 450 and scanned using a Gene Chip Scanner 3000 7G.

### Extracellular vesicle transfer experiments

EVs were collected from SEN and NSEN myoblasts via ultracentrifugation, as described above. After EV isolation, a small fraction was lysed to determine the protein concentration, and samples were normalized to have the same approximate number of particles (2 × 10^8^ particles/mL). Myoblasts cultured in GM-EV were then treated with the isolated NSEVs or SEVs every 24h for a total of 48h before being collected for RT-qPCR. NSEV-T and SEV-T designate myoblasts after NSEV treatment and SEV treatment, respectively.

### RNA-Seq data sets of mouse gastrocnemius muscle

The gastrocnemius muscle of male C57BL/6JRj mice at 8 months-old (8mo; *n=*8) and 28 months-old (28mo; *n=*9) was collected and sequenced as part of a previous study [26]. RNA-Seq data were obtained from the Gene Expression Omnibus (GEO; https://www.ncbi.nlm.nih.gov/geo/) under the accession number GSE145480. Data were filtered to remove other time points, and significance was set at a false discovery rate (FDR) < 0.05 and fold change ≥ |1.5| to identify differentially expressed genes (DEG). Functional enrichment analysis using the Kyoto Encyclopedia of Genes and Genomes (KEGG) database was performed on DEG that were also validated gene targets of 2+ SEV miRNAs. Data processing and visualization were executed as stated below.

### Data processing and statistical analysis

### Microarray data analysis

After the miRNA 4.0 arrays were scanned, the raw intensities in a CEL format were exported and subsequent data processing and analysis were performed in RStudio with R version 4.3.2. CEL files and the sample and data relationship format (SDRF) file were read into R using the *oligo* package [57], and chip quality control was evaluated through the *arrayQualityMetrics* package [58] and raw intensity/pseudoimage chip visualization. MiRNA expression was preprocessed using robust multichip averaging (RMA), which performs background subtraction, quantile normalization, and summarization of probe-level data [59]. Low intensity probes were filtered out, and probe annotation was performed using the miRNA 4.0 annotations file (Affymetrix). Only mouse miRNAs were filtered in, and other species or small RNA types were removed prior to model fitting. Subsequently, linear modelling was performed to find the differentially expressed (DE) mouse miRNAs between SEN and NSEN-derived EVs using the *limma* package [60]. Multiple test corrections were applied using the Benjamini-Hochberg method to estimate the FDR, and we set significance thresholds to an FDR < 5% and fold change ≥ |1.5| to determine DE miRNAs [61]. For cross-species validation of DE mouse miRNAs (Supplementary Figure 1), additional miRNAs from other species that contained a nucleotide sequence not found in any mouse miRNA were added to the analysis. The *multiMiR* package was employed to identify validated targets of upregulated and downregulated miRNAs from several external databases (miRTarBase, miRecords, and TarBase) [62]. Identified gene targets were filtered to remove replicates before undergoing gene ontology (GO) analysis and functional enrichment analysis using the KEGG database through the *clusterProfiler* package [63]. GO and KEGG plots were ordered by adj. p and filtered for related terms. GO biological process network mapping was generated from the *multiMiR*-identified gene targets after reducing redundant GO terms using the simplify function of the *clusterProfiler* package and filtering for terms with FDR < 5 x10^-5^. Upset plots were generated with the *UpSetR* package using discrete upregulated and downregulated miRNA targets, and plots were filtered to show intersections between 2 or more miRNAs to best display overlaps in miRNA regulation of gene targets [64]. The MiRNet 2.0 web tool (https://www.mirnet.ca) was used to generate a network visual between all DE miRNAs and their target genes [65]. The degree filter was set to a cutoff of 5 to limit network size and display only the gene targets that connected to more than 5 miRNAs. Cytoscape (V3.10.2) was then used to visualize the miRNA-gene interaction network. The probability of gene list overlap was calculated with a hypergeometric distribution. A full list of the packages used for RNA-Seq and miRNA microarray data processing and analysis can be found in the supplementary file (Supplementary Table 1).

### Experimental data analysis

For the statistical comparison of two groups, unpaired two-tailed Student’s t-tests were employed using RStudio with R version 4.3.2. Normality was evaluated with the Shapiro-Wilk test, and in the case of non-normal data, a non-parametric test was utilized. Statistical significance was accepted at *p* < 0.05, and significance symbols are defined in figure captions. Data are presented as means ± SD. Bar graphs were generated with Prism (V9.5.1, Graphpad Software LLC).

### Data availability

The data that support the findings of this study are openly available in Gene Expression Omnibus (GEO) at https://www.ncbi.nlm.nih.gov/geo/, reference number GSE297375.

## Supplementary Material

Supplementary Figures

Supplementary Table 1
