# Supplementary material for: Extracellular vesicles released by senescent myoblasts affect recipient cells via miRNA-target interactions
Source: Aging (Albany NY). 2026 May 15;18(1):531–53. doi: 10.18632/aging.206379 (PMC13632851; doi:10.18632/aging.206379)
Supplement: Supplementary file 1 [file aging-18-1-206379-s001.pdf]

SUPPLEMENTARY FIGURES

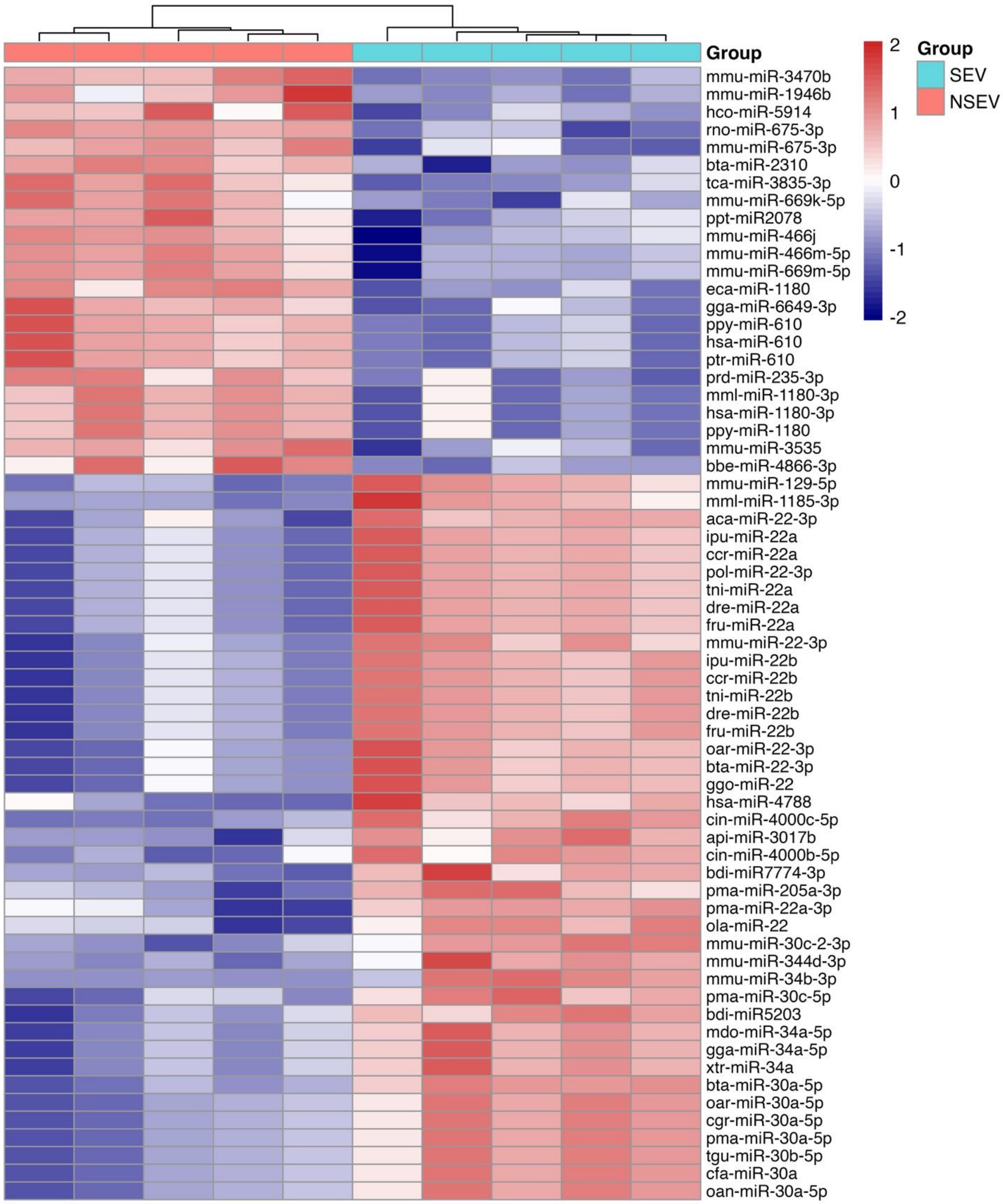

Supplementary Figure 1. Heatmap of DE miRNAs across all species. In addition to mouse miRNAs, other species were included prior to model fitting. Other species' miRNAs were removed if their nucleotide sequence was found in any mouse miRNA. Prefixes indicate species designations (*i.e.,* "mmu" for "*Mus musculus*" [mouse], "hsa" for "*Homo sapiens*" [human], etc.).

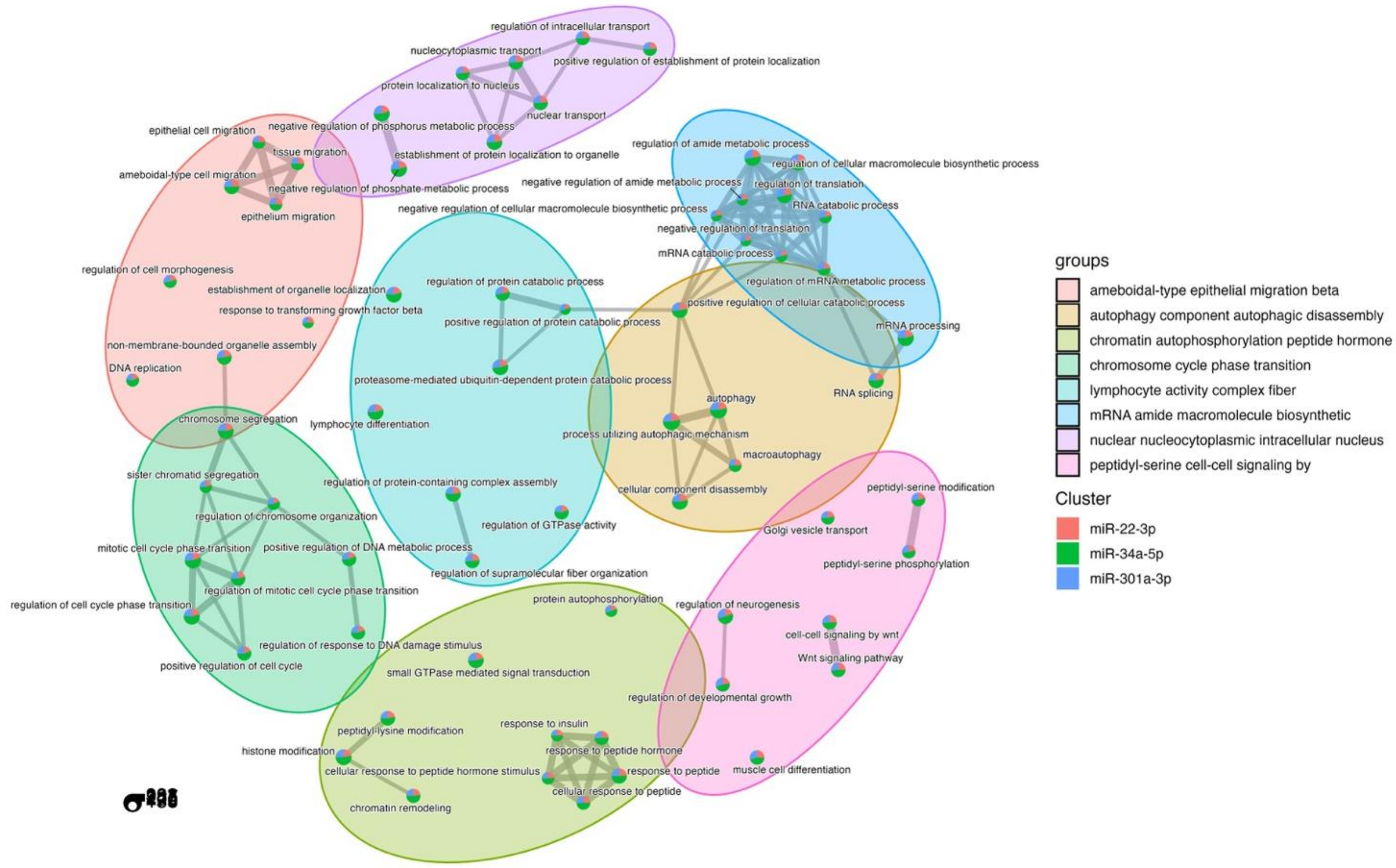

Supplementary Figure 2. Network mapping of the top GO biological pathways that overlap between the gene targets of miR-34a, miR-22, and miR-301a. GO terms were clustered into groups and compared between miRNA clusters using the R function *compareCluster* in the *clusterProfiler* package. The top 35 significant (q < 0.05) GO-BP ontologies are displayed and grouped based on overlap. Pie-chart nodes indicate when genes targeted by each miRNA contribute to that term.

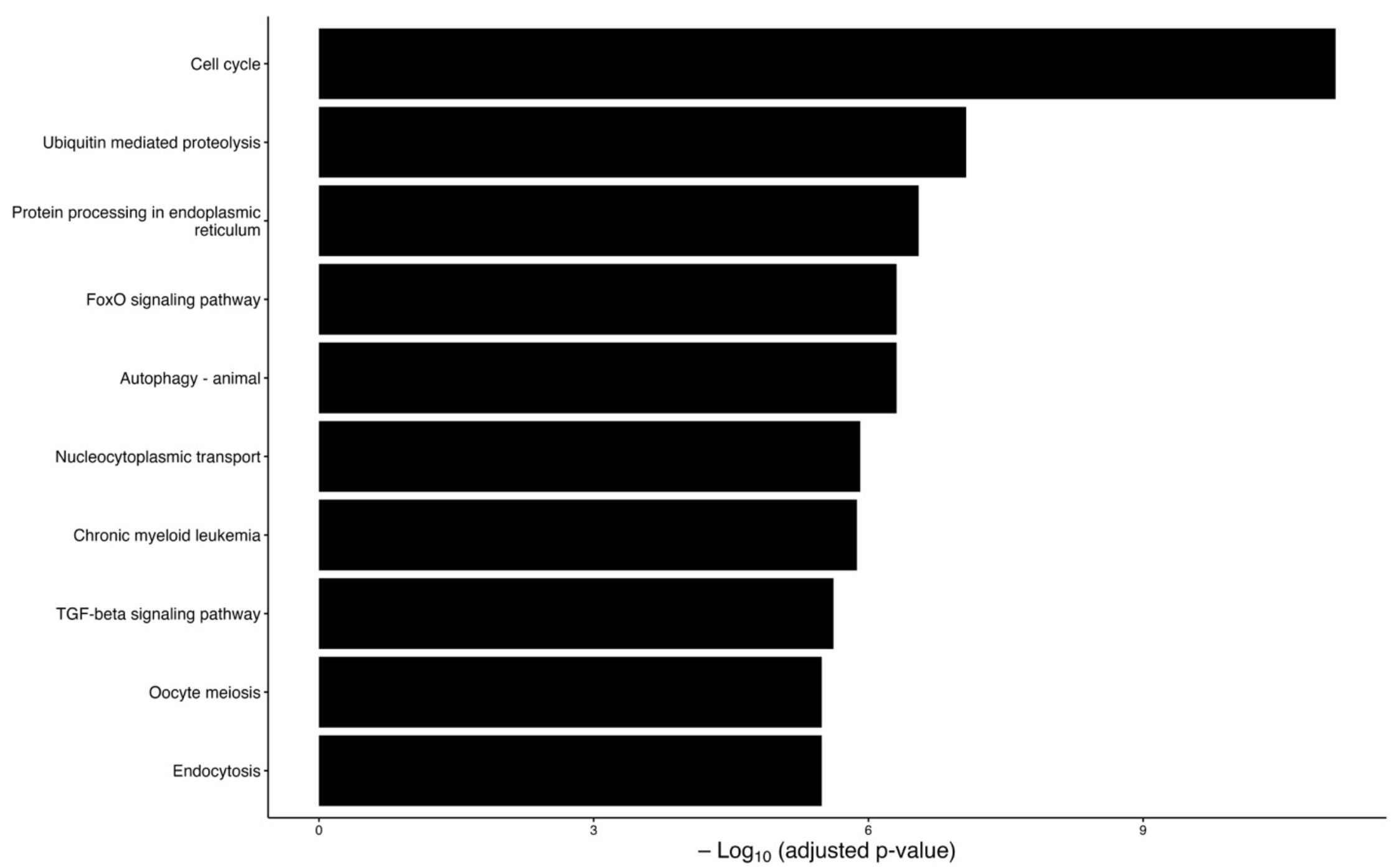

Supplementary Figure 3. KEGG enrichment analysis of miR-301a-3p gene targets.

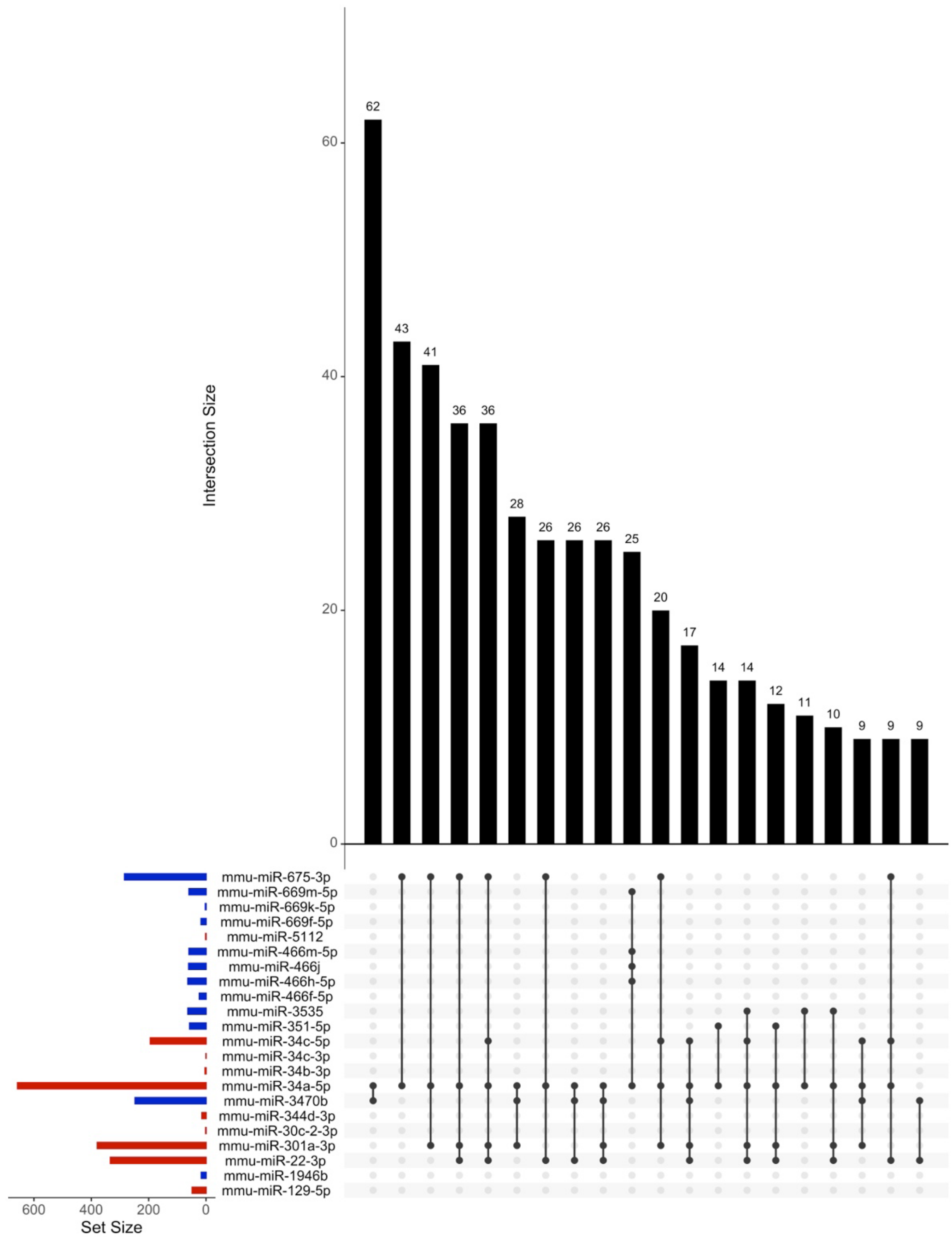

Supplementary Figure 4. Upset plot depicting the number of shared gene targets between divergently expressed miRNAs. Plot has been filtered to remove intersections exclusively between miRNAs that changed in the same direction. Red set bars highlight upregulated miRNAs, and blue set bars highlight downregulated miRNAs.
