# Supplementary material for: Extracellular vesicles released by senescent myoblasts affect recipient cells via miRNA-target interactions
Source: Aging (Albany NY). 2026 May 15;18(1):531–53. doi: 10.18632/aging.206379 (PMC13632851; doi:10.18632/aging.206379)
Supplement: Supplementary file 2 [file aging-18-1-206379-s002.pdf]

SUPPLEMENTARY TABLE

Supplementary Table 1. R packages.

Package name	Role	Version
oligo	Pre-processing tools	1.70.0
arrayQualityMetrics	Chip quality control	3.62.0
ggplot2	Graph creation	3.5.1
Biobase	Base functions for Bioconductor	2.66.0
limma	Linear models for microarray data	3.62.1
tidyr	2.88	1.3.1
dplyr	2.61	1.1.4
stringr	2.60	1.5.1
pheatmap	Heatmap visualization	1.0.12
EnhancedVolcano	Volcano plot visualization	1.24.0
multiMiR	DE miRNA gene target identification	1.28.0
clusterProfiler	Analysis and visualization of gene enrichments	4.14.3
UpSetR	Upset plot intersections and visualization	1.4.0
pathview	Pathway data visualization	1.46.0
org.Mm.eg.db	Genome wide annotation for Mouse	3.20.0
enrichplot	Visualization of enrichment plots	1.27.2
PCAtools	PCA visualization	2.18.0
pd.mirna.4.0	Platform design for miRNA 4.0	3.12.0
AnnotationDbi	Manipulation of SQLite Annotations	1.68.0
